# Results Obtained from the Use of Anti-Dysenteric Sero-Vaccine in the Field with Regard to the Reduction of Case Incidence

**Published:** 1918-05

**Authors:** 


### ABSTRACTS

### DYSENTERY

***Results Obtained from the Use of Anti-Dysenteric Sero-Vaccine in the Field with Regard to the Reduction of Case Incidence.*** By Major H. Graeme Gibson, R. A. M. C., Preliminary Notice of an Article to Appear in the J. R. A. M. C.

The present article is a report of trials in the use of vaccine previously announced.

The tests were arranged in two spheres of operation (the Near East and the Western Front) and in each case the incidence of bacillary dysentery was diminished among the men inoculated. Although the total incidence of dysentery was not high on the Eastern Front, there were each year two rises in the curve of incidence among the troops, one at the commencement of hot weather and one in the fall.

The men inoculated were given two doses of sero-vaccine towards the end of the first rise in the curve. The laboratory officer reported that the inoculation had conferred a considerable measure of immunity. There were no ill effects from the inoculations, except for a considerable reaction on the first day, which, however, soon passed off. There were some slight local reactions for about forty-eight hours.

The number of men inoculated was 1150. There were 1550 controls.

Among the inoculated men, 9 cases of dysentery occurred, and more among men who had received only one dose. During the same period there were 66 cases of bacillary dysentery among the uninoculated. Of the 9 cases among the inoculated, 7 occurred within 4 weeks of the inoculation and only 2 at a later time. The men were observed for a period of 14 weeks.

In a base camp in France 975 men were inoculated and 1361 kept as controls. There were 4 cases of dysentery among the inoculated and 14 cases among the controls.

There was no agglutinin demonstrable in the serum after the

1. *J. R. A. M. C.*, June, 1917

first dose of vaccine when tested with B. Shiga antigen, and very little with B. dysenteriae Flexner and B. dysenteriae Y antigens.

The agglutinins in the serum do not appear to reach their highest point until three weeks after the second dose. Titers taken at the end of ten weeks are as good if not better than those at the end of three weeks. Although there is no negative phase, it appears that a long latent period precedes the body's response to the vaccine. The writer quotes Castellani to the effect that agglutinins develop for all the organisms of the dysentery group, but that they are seldom higher than 1-40. They are inconstant and irregular.

It has been found more difficult to confer immunity against this group of diseases than against the enteric group. Unless there is some agglutinin present in the blood, there is little evidence of protection. A patient who developed dysentery due to B. Shiga, although he had been inoculated, failed to show any agglutinins at the time he contracted the disease.

Immunity lasts probably from four to six months.

The writer does not recommend wholesale prophylactic inoculation for troops, but he thinks it would be well to give preventive injections at the beginning of the summer to troops among whom there has been an epidemic of dysentery and hence the possibility of carriers. Inoculation would also be helpful, he believes, in a unit in which there is a sudden outbreak of the disease.

